# Identification of Angiogenic Cargo in Extracellular Vesicles Secreted from Human Adipose Tissue-Derived Stem Cells and Induction of Angiogenesis In Vitro and In Vivo

**DOI:** 10.3390/pharmaceutics13040495

**Published:** 2021-04-05

**Authors:** Prakash Gangadaran, Ramya Lakshmi Rajendran, Ji Min Oh, Eun Jung Oh, Chae Moon Hong, Ho Yun Chung, Jaetae Lee, Byeong-Cheol Ahn

**Affiliations:** 1 BK21 FOUR KNU Convergence Educational Program of Biomedical Sciences for Creative Future Talents, Department of Biomedical Sciences, School of Medicine, Kyungpook National University, Daegu 41944, Korea; prakashg@knu.ac.kr (P.G.); hy-chung@knu.ac.kr (H.Y.C.); 2 Department of Nuclear Medicine, School of Medicine, Kyungpook National University, Daegu 41944, Korea; ramyag@knu.ac.kr (R.L.R.); ojm0366@knu.ac.kr (J.M.O.); jaetae@knu.ac.kr (J.L.); 3 Department of Plastic and Reconstructive Surgery, CMRI, School of Medicine, Kyungpook National University Hospital, Kyungpook National University, Daegu 41944, Korea; fullrest74@knu.ac.kr; 4 Department of Nuclear Medicine, School of Medicine, Kyungpook National University Hospital, Kyungpook National University, 680 Gukchaebosangro, Junggu, Daegu 41944, Korea; cmhong@knu.ac.kr

**Keywords:** adipose tissue, stem cell, extracellular vesicle, angiogenesis

## Abstract

Angiogenesis is defined as the generation of new blood vessels or the sprouting of endothelial cells from a pre-existing vascular network. Angiogenesis occurs during the growth and development of an organism, the response of organs or tissues to injury, and during cancer development and progression. The majority of studies on stem-cell-derived extracellular vesicles (EVs) have used cell lines, and have primarily focused on well-known solitary proteins. Here, we isolated stem cells from human adipose tissue (ADSCs), and we isolated EVs from them (ADSC-EVs). The ADSC-EVs were characterised and 20 angiogenic proteins were analysed using an angiogenic antibody array. Furthermore, we analysed the ability of ADSC-EVs to induce angiogenesis in vitro and in vivo. ADSC-EVs were positive for CD81 and negative for GM130, calnexin, and cytochrome-C. ADSC-EVs showed typical EV spherical morphology and were ~200 nm in size. ADSC-EVs were found to contain angiogenic proteins as cargo, among which interleukin 8 (IL-8) was the most abundant, followed by chemokine (C-C motif) ligand 2 (CCL2), a tissue inhibitor of metalloproteinases 1 (TIMP-1), TIMP-2, and vascular endothelial growth factor-D (VEGF-D). ADSC-EVs treatment increased the proliferation, migration, total vessel length, total number of junctions, and junction density of endothelial cells in vitro. The results of an in vivo Matrigel plug assay revealed that ADSC-EVs induced more blood vessels in the Matrigel compared with the control. These results demonstrate that ADSC-EVs contain angiogenic proteins as cargo and promote angiogenesis in vitro and in vivo. Therefore, ADSC-EVs have potential for therapeutic use in ischaemia.

## 1. Introduction

Angiogenesis is described as the generation of new blood vessels or the sprouting of endothelial cells from a pre-existing vascular network. Angiogenesis occurs naturally during the growth and development of an organism. Ischaemia, a restriction in blood supply to tissues, deprives cells and tissues of oxygen, which is required for cellular metabolism [1]. Angiogenesis also occurs during the response to ischaemia, to restore blood supply to the ischaemic tissues [2]. Blood vessel growth is initiated by an imbalance in the expression of angiogenic factors as a result of pathological conditions [3,4,5]. Ischaemic diseases are some of the deadliest, and account for millions of deaths worldwide [6,7]. Therefore, the development of novel therapeutic strategies for the treatment of ischaemic diseases is urgently needed.

Many preclinical and clinical studies have reported therapeutic effects of stem cells in ischaemic brain injury, ischaemic heart failure, and limb ischaemia [8,9,10,11,12,13]. Although stem cell therapies have been shown to have numerous advantages over conventional therapies, their feasible translation is hindered by many factors, including the isolation of stem cells (especially stem cells derived from the bone marrow, embryo, and umbilical cord), the inability to control differentiation after administration, immunological rejection, tumour formation, inappropriate stem cell migration, the viability of cells after administration, and the limited understanding of how stem cells work in vivo [14,15,16,17,18,19]. Therefore, it is important to develop other therapeutic approaches to accelerate angiogenesis in ischaemia with no or minimal adverse effects.

It has recently been accepted that the therapeutic effects of many cells, including stem cells, occur primarily through the secretion of paracrine factors, including extracellular vesicles (EVs) [20,21,22,23,24]. EVs are nano-sized vesicles secreted by almost all cells, and consist of exosomes and small EVs (30–200 nm), as well as micro-vesicles (30–500 nm). EVs are capable of carrying functionally active biological materials such as lipids, proteins, mRNAs, miRNAs, and DNAs, and delivering them to recipient cells [25,26]. Given their biological properties, the exploitation of EVs as therapeutic agents is a primary research focus.

Numerous studies have recently shown that EVs derived from mesenchymal stem cells (MSC-EVs) are capable of inducing angiogenesis in the context of ischaemic diseases and wound healing [20,23,24,27,28,29,30,31]. However, most of these previous studies used EVs derived from either cell lines or bone marrow stem cells, while others used EVs derived from umbilical cord stem cells. However, few studies have isolated EVs from primary human adipose tissue-derived stem cells (ADSCs) to investigate their angiogenic potential [32]. ADSCs are easily and readily isolated from adipose tissues that are discarded during liposuction procedures or other surgeries; however, bone marrow stem cells are not readily available, and isolation requires complex procedures [33,34]. The most important consideration in EV isolation is the number of cells required to produce a sufficiently large number of EVs to be used clinically or for exclusive personalised therapies (autologous or allogeneic). Previous studies have reported that stem cells isolated from adipose tissues give rise to 500 times more cells compared with those from bone marrow aspirates [34,35].

Here, we isolated ADSC-EVs from ADSCs harvested from human tissues to examine the presence of angiogenic protein cargo in ADSC-EVs, and to investigate the angiogenic effects of ADSC-EVs both in vitro and in vivo.

## 2. Materials and Methods

### 2.1. Collection of ADSCs and Cell Culture

Adipose tissues were obtained from discarded subcutaneous tissue during flap surgery, as described previously [36]. ADSCs and SVEC-4 (mouse endothelial cell line) were cultured in DMEM (Gibco, Carlsbad, CA, USA) supplemented with 10% foetal bovine serum (FBS; Hyclone, Logan, UT, USA). Both cell types were supplemented with 10% EV-depleted FBS (the FBS was ultracentrifuged for 18 h at 120,000× *g* at 4 °C) and 1% penicillin and streptomycin, in a CO_2_ incubator at 37 °C.

### 2.2. Isolation of EVs

**Figure 1:**
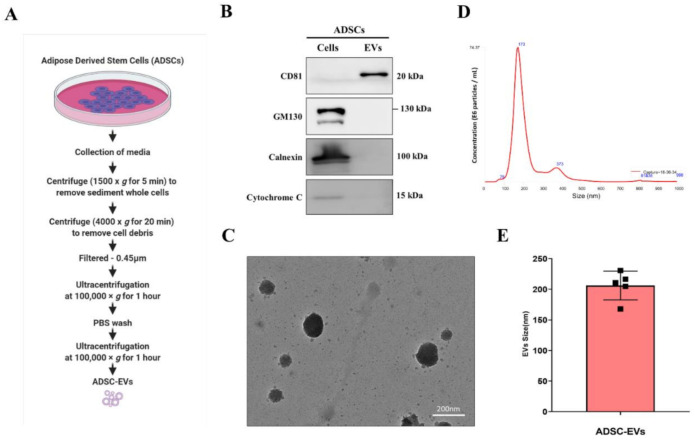
Isolation and characterisation of adipose tissue-derived stem cell extracellular vesicles (ADSC-EVs). (**A**) A schematic diagram demonstrating the isolation of EVs from ADSCs (created with BioRender.com). (**B**) Western blot analysis of ADSCs (cells and EVs); anti-CD81, anti-GM130, calnexin, and anti-cytochrome-C antibodies were used. (**C**) The morphology of ADSC-EVs visualised by TEM (scale bar: 200 nm). (**D**,**E**) NTA of ADSC-EVs (*n* = 5). The results are expressed as the mean ± SD values of the experiment.

ADSC cultured media (up to five passages) were collected and EVs were isolated as illustrated in Figure 1A. The supernatants were briefly centrifuged for 10 min at 1500× *g* to remove the cells, and then for 20 min at 4000× *g* to remove the cell debris. The collected supernatant was filtered through a 0.45 µm syringe. The samples were ultracentrifuged for 60 min at 100,000× *g* The collected pellets were washed with phosphate-buffered saline (PBS) and again ultracentrifuged for 60 min at 100,000× *g* The pellets were reconstituted in 50–100 µL PBS and stored at −80 °C until use. All ultracentrifugation procedures were performed using a SW28 rotor at 4 °C (Beckman Coulter, Brea, CA, USA). The concentration of EVs was measured with a Pierce BCA Protein Assay Kit (Thermo Fisher Scientific, Waltham, MA, USA).

### 2.3. Western Blotting

Western blot analysis was performed as described in a previous study [22]. ADSC and ADSC-EVs lysates were prepared in RIPA buffer (Thermo Fisher Scientific, Waltham, MA, USA). Equal amounts of protein were loaded and separated using 10% SDS-polyacrylamide gel electrophoresis. The proteins were transferred to PVDF membranes (Millipore, Burlington, MA, USA). The blots were first probed with the primary antibody (CD81 (1:2500), Alix (1:2500), and cytochrome-C (1:5000); all Abcam), and then with the secondary antibody conjugated with horseradish peroxidase (Cell Signalling Technology, Danvers, MA, USA). The signals were detected using enhanced chemiluminescence (GE Healthcare, Chicago, IL, USA) according to the manufacturer’s instructions.

### 2.4. Transmission Electron Microscopy (TEM)

The ADSC-EVs were resuspended in 2% paraformaldehyde (100 µL). ADSC-EVs pellets were attached to Formvar/carbon-coated EM grids. To avoid exposure to light, the sample was covered with aluminium foil for 20 min to avoid any damage/dryness to the sample. The sample was washed with 100 µL PBS and incubated in 1% of glutaraldehyde for 5 min. The sample was washed with distilled water seven times (2 min each) and viewed under HT 7700 TEM to observe the size of the ADSC-EVs [20].

### 2.5. Nanoparticle Tracking Analysis (NTA)

The size of the ADSC-EVs was determined by NTA using a NanoSight LM10 (Malvern) with a 640 nm laser according to the described protocol. ADSC-EVs were briefly diluted in Milli-Q water (1:1000) and the sample was injected into the chamber using a sterile syringe. The measurements were performed and the average of the results was recorded [20].

### 2.6. Human Angiogenesis Array

Proteins extracted from ADSC-EVs (150 µg/blot) of two human subjects were used for this assay. The Human Angiogenesis Array (RayBiotech, Peachtree Corners, GA, USA) was used according to the manufacturer’s instructions. The intensity was measured by GelQuant.NET software (Version 1.8.2) (BiochemLabSolutions.com, San Francisco, CA, USA).

### 2.7. EV Labelling and Internalisation Assay

The internalisation of ADSC-EVs was analysed by confocal microscopy. The EVs were labelled with lipophilic dye (DiD) by incubating for 20 min at 37 °C and washing with PBS by ultracentrifugation, as mentioned above. The DiD-labelled ADSC-EVs (5 or 10 µg/mL) or unlabelled ADSC-EVs (5 or 10 µg/mL) were incubated with endothelial cells for 2 h at 37 °C before methanol fixation. Antifade agent was used to mount the coverslips (Vector Laboratories, Burlingame, CA, USA). The uptake of ADSC-EVs was observed by LSM 800 laser scanning microscopy (Carl Zeiss, Baden-Württemberg, Germany).

### 2.8. In Vitro Transwell Migration Assay

First, 600 μL of medium with 3% FBS was added into the lower chamber of a 24-well plate. Then, endothelial cells (1.5 × 10^4^) in 300 μL medium (0% FBS) was added onto the transwell membrane insert. Immediately, ADSC-EVs (5 and 10 μg/mL) were added onto the top of the insert and incubated for 24 h in a CO_2_ incubator. The cells were fixed and stained with crystal violet as previously described [37]. The cells were visualised and imaged with an AXIO microscope (Zeiss, Baden-Württemberg, Oberkochen, Germany).

### 2.9. In Vitro Cellular Proliferation Assay

Endothelial cells were seeded at 1 × 10^4^ cells/well in 100 μL of medium in a 96-well plate, and incubated in a CO_2_ incubator overnight. Then, the cells were treated with 0, 5, 10, 15, or 20 μg/mL of ADSC-EVs for 24 h. Cellular proliferation was assessed by the optical density at a 450 nm wavelength using a spectrophotometer, according to the manufacturer’s protocol (CCK8 assay kit, Dojindo Molecular Technologies, Kyushu, Japan), and the results are presented as percentages.

### 2.10. In Vitro Matrigel Tube Formation Assay

Matrigel Growth Factor Reduced Basement Membrane Matrix (Conc: 100%) (Matrigel; Corning) was coated onto 24-well culture plates followed by incubation at 37 °C for 1 h to allow the gel to settle. Then, 2 × 10^4^ endothelial cells/well were added into the plates followed by ADSC-EVs (5 and 10 μg/mL). After 4 h, the cells were visualised and imaged (four images/group) with fluorescence microscopy (AXIO, Zeiss, Oberkochen, Germany). The total vessel length, number of junctions, and density of junctions were counted/measured automatically (*n* = 4) by AngioTool64 software (National Cancer Institute, Radiation Oncology Branch, Angiogenesis Core Facility, Maryland, MD, USA) [38].

### 2.11. In Vivo Matrigel Plug Assay in Mice

The mice (*n* = 7) were anaesthetised, and the following mixture was subcutaneously injected into the right lower flank. The Matrigel-only group mice (*n* = 3) were administered 300 μL Matrigel (Conc. 100%) and the Matrigel + ADSC-EVs group mice (*n* = 4) were administered 300 μL Matrigel (Conc. 100%) + 50 μg ADSC-EVs. The Matrigel plugs were removed after 2 weeks, and the images were captured using a surgical microscope (M320 F12; Leica Microsystems, Germany).

### 2.12. Histology and Microscopy

The Matrigel plugs were fixed immediately after imaging in 10% neutral buffered formalin, embedded in paraffin, and sectioned at 3–4 μm. The sections were stained with haematoxylin and eosin (H&E) [8]. The H&E slide sections were imaged under an AXIO microscope (Zeiss, Oberkochen, Germany). The number of blood vessels per image or plug was counted.

### 2.13. Statistical Analysis

All data are expressed as the mean ± standard deviation (SD). Two groups of data were analysed by Student’s *t*-test in MS Office Excel sheet (Microsoft, Redmond, WA, USA) or GraphPad Prism 9 software version 9.0.0 (GraphPad Software, Inc., San Diego, CA, USA). A *p*-value < 0.05 was considered statistically significant.

## 3. Results

### 3.1. Characterisation of ADSC-EVs

The primary ADSC cultured media was collected and used for the isolation and purification of ADSC-EVs by ultracentrifugation, as illustrated in Figure 1A. The enrichment and purity of EVs were confirmed by the well-characterised positive and negative markers of ADSC-EVs. Western blotting results showed an enrichment of CD81 (a membrane protein) in the ADSC-EVs fraction compared with the source cells; whereas the negative markers, GM130 (Golgi apparatus), calnexin (endoplasmic reticulum), and cytochrome-C (mitochondria), were only present in the cells (Figure 1B). These results confirmed that ADSC-EVs isolated from ADSCs were intact and without cellular contamination. The morphology of ADSC-EVs was observed by TEM, which revealed that the EVs had a typical spherical morphology and were mostly intact (Figure 1C). The size of the ADSC-EVs was confirmed by NTA, which revealed a single enriched peak at 178 nm and an average diameter of 206.2 ± 23.5 nm (Figure 1D,E). These results suggest that ADSC-EVs were successfully isolated from human primary ADSCs.

### 3.2. Angiogenic Protein Cargo in Human ADSC-EVs

**Figure 2:**
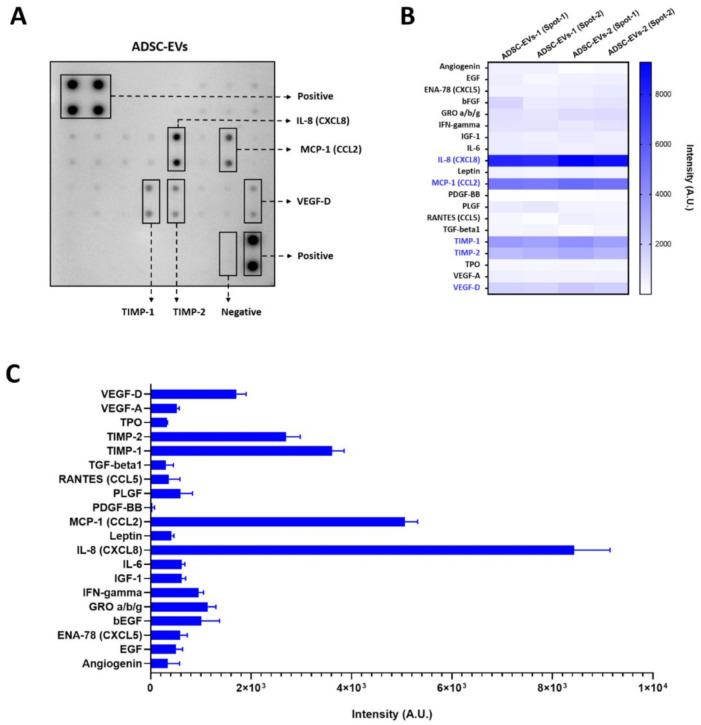
Angiogenic array reveals the presence of angiogenic proteins in ADSC-EVs. (**A**) The representative array blot incubated with ADSC-EVs lysate (150 µg/blot). (**B**) The quantitative results of the spots of each protein (*n* = 4). (**C**) Bar graph of the quantitative results (intensity) of the spots of each protein (*n* = 4).

Twenty angiogenic proteins in the EVs isolated from ADSCs were analysed by angiogenic array (Figure 2A). The results revealed that interleukin 8 (IL-8), also called chemokine (C-X-C motif) ligand 8 (CXCL8), was present in ADSC-EVs at a higher concentration than the other proteins, followed by chemokine (C-C motif) ligand 2 (CCL2), a tissue inhibitor of metalloproteinases 1 (TIMP-1), TIMP-2, and vascular endothelial growth factor-D (VEGF-D) (Figure 2B,C). Growth-regulated oncogene alpha/beta/gamma (GRO α/β/γ), basal fibroblast growth factor (bFGF), and interferon gamma (IFN-γ) were also present at low levels. The remaining proteins were present at low levels, and the level of platelet-derived growth factor BB was negligible (Figure 2B,C). These results clearly confirm that the ADSC-EVs were packed with various angiogenic proteins.

### 3.3. Endothelial Cell Internalisation of ADSC-EVs

**Figure 3:**
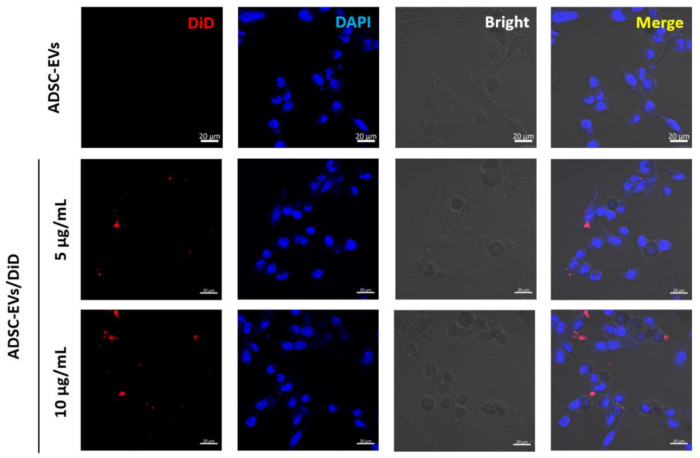
Endothelial cells internalised ADSC-EVs. Endothelial cells were incubated with unlabelled ADSC-EVs (10 µg/mL) and DiD-labelled ADSC-EVs (5 or 10 µg/mL; ADSC-EVs/DiD) for 2 h; arrows indicate internalised ADSC-EVs (scale bar: 20 µm).

The internalisation of ADSC-EVs by endothelial cells was confirmed by fluorescence microscopy, by labelling the ADSC-EVs with lipophilic dye (DiD). The results reveal that ADSC-EVs were readily internalised by endothelial cells (Figure 3).

### 3.4. Human ADSC-EVs Promote the Migration and Proliferation of Endothelial Cells

**Figure 4:**
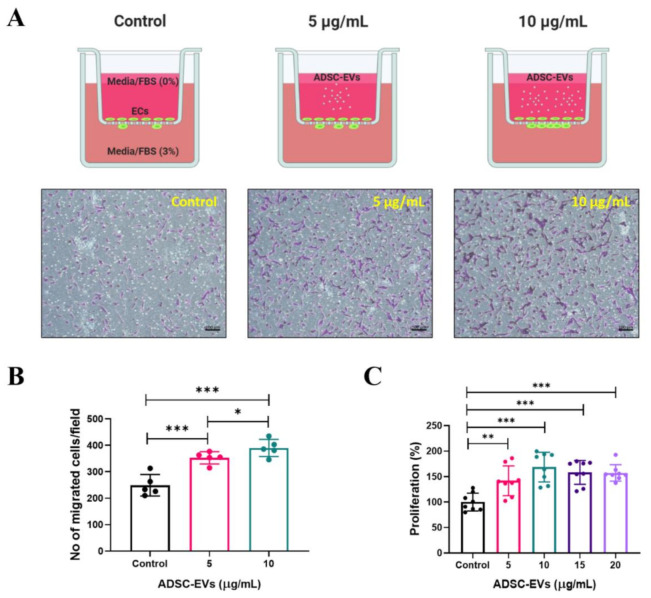
Increased migration and proliferation of endothelial cells by treatment with ADSC-EVs. (**A**) Graphical scheme of the migration assay (figure created with BioRender.com) and phase contrast microscopy images of migrated endothelial cells after 24 h treatment with ADSC-EVs (5 and 10 µg/mL). (**B**) Quantified data of migrated cells shown in (**A**) (*n* = 5). (**C**) Graph showing the proliferation of endothelial cells quantified via CCK8 assay after 24 h treatment with 0–20 µg/mL of ADSC-EVs (*n* = 8). * *p* < 0.05, ** *p* < 0.01, *** *p* < 0.001 by Student’s *t*-test.

Angiogenesis is a complex multistep process, wherein endothelial cells migrate, proliferate, and generate new blood vessels. The ability of ADSC-EVs to induce the migration of endothelial cells was examined. The transwell migration results showed that ADSC-EVs (5 and 10 µg/mL) were able to significantly (*p* < 0.001) increase the migration of endothelial cells, which was further increased by treatment with 10 µg/mL of ADSC-EVs (*p* < 0.05). Thus, endothelial cell migration was increased by ADSC-EVs in a dose-dependent manner (Figure 4A,B). Next, the ability of ADSC-EVs (5, 10, 15, and 20 µg/mL) to induce the proliferation of endothelial cells was investigated. The results showed that the proliferation of endothelial cells significantly increased following treatment with ADSC-EVs (5 µg/mL: *p* < 0.01 and 10–20 µg/mL: *p* < 0.001) compared with the control (Figure 4C). These results show that ADSC-EVs are capable of inducing the migration and proliferation of endothelial cells.

### 3.5. ADSC-EVs Promote Tube Formation in Endothelial Cells

**Figure 5:**
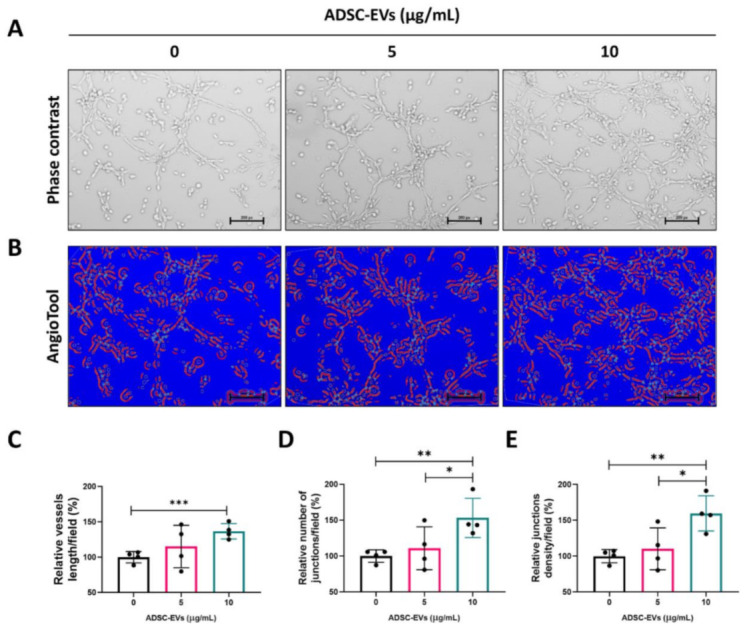
Treatment with ADSC-EVs promoted the in vitro tube-like formation of endothelial cells. (**A**) Representative phase contrast images showing endothelial cells cultured on Matrigel-coated plates in medium with ADSC-EVs (0, 5, or 10 μg/mL) (scale bar: 200 pixels). (**B**) AngioTool64 software was used to analyse the images, and the vessels (red lines) and junctions (blue dots) are shown. (**C**–**E**) The relative total vessel length, number of junctions, and junction density were measured in the images (*n* = 4) using AngioTool64 software, Version 0.6a (02.18.14). * *p* < 0.05, ** *p* < 0.01, *** *p* < 0.001 by Student’s *t*-test.

Next, we tested the capacity of endothelial cells to form blood vessels in vitro following treatment with ADSC-EVs (5 and 10 µg/mL) for 4 h. Following treatment, the endothelial cells were imaged with a microscope (Figure 5A) and the images were analysed by AngioTool software (National Cancer Institute, Radiation Oncology Branch, Angiogenesis Core Facility, Maryland, MD, USA). The images represent the vessel length, number of junctions, and junction density (Figure 5B; redline tube-like structure and red dot: junctions). The vessel length was substantially increased at 5 µg/mL, and significantly increased (*p* < 0.001) at 10 µg/mL compared with the control (Figure 5C). The number of junctions was substantially increased at 5 µg/mL compared with the control, and significantly increased (*p* < 0.05 and *p* < 0.01) at 10 µg/mL compared with 5 µg/mL and the control (Figure 5D). The density of the junctions was also substantially increased at 5 µg/mL compared with the control and significantly increased (*p* < 0.05 and *p* < 0.01) at 10 µg/mL compared with 5 µg/mL and the control (Figure 5E). These results confirm that ADSC-EVs are capable of inducing angiogenesis.

### 3.6. ADSC-EVs Promote Angiogenesis In Vivo

**Figure 6:**
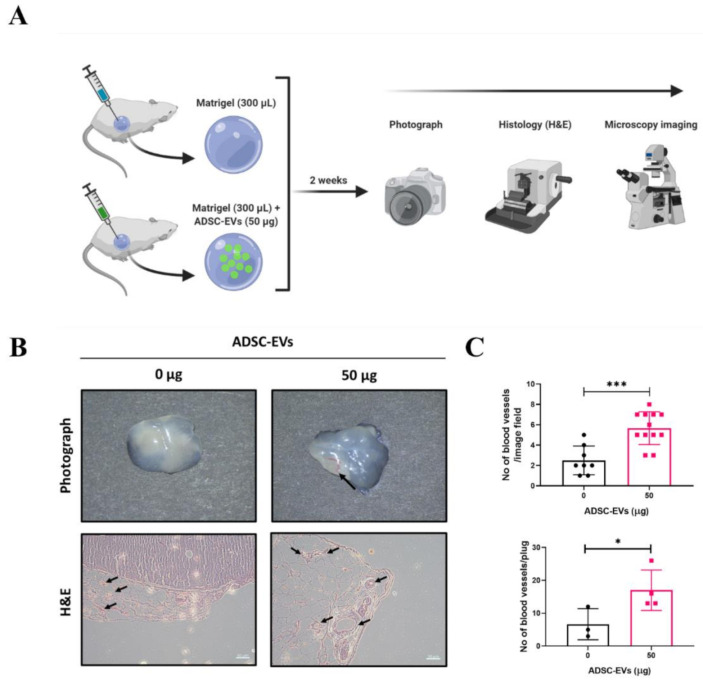
Treatment with ADSC-EVs increased the number of blood vessels in a Matrigel plug mouse model. (**A**) A schematic diagram of the in vivo Matrigel plug mouse model (figure created with BioRender.com). (**B**) Representative photograph and H&E staining of the Matrigel plugs for the control and Matrigel + ADSC-EVs (50 μg) groups; black arrows denote blood vessels (scale bar: 50 μm). (**C**) The number of blood vessels was counted from the images of H&E staining. * *p* < 0.05, *** *p* < 0.001 by Student’s *t*-test.

After confirming the in vitro angiogenic effect (blood-vessel-like formation) of ADSC-EVs, the in vivo angiogenic effect was tested in a mouse model. A schematic diagram of the in vivo Matrigel plug mouse model is presented in Figure 6A. Matrigel (*n* = 3) or Matrigel + ADSC-EVs (*n* = 4) was subcutaneously injected into mice. The Matrigel plugs were removed after 2 weeks and were visually examined and photographed using a surgical microscope. The Matrigel plugs of the Matrigel group showed minimal or no blood vessels, but the Matrigel plugs of the Matrigel + ADSC-EVs group showed blood vessels (upper panel; Figure 6B). Cross sections of the Matrigel plugs were stained with H& E, and the results confirmed the presence of blood vessels in both groups (lower panel; Figure 6B). The number of blood vessels in the Matrigel plugs of the Matrigel + ADSC-EVs group was significantly greater than the number in the control (*p* < 0.001: per image field and *p* < 0.05 per plug) (Figure 6C). These results confirm that ADSC-EVs are capable of inducing angiogenesis in vivo.

## 4. Discussion

Angiogenesis is essential for recovery from ischaemic diseases. Stem-cell-derived EVs have recently emerged as a promising therapeutic candidate for numerous diseases [21,29,32,39], but the clinical translation of stem-cell-derived EVs has not yet been achieved. In this study, we aimed to isolate stem-cell-derived EVs with the potential to be used in clinical translation. To this end, we isolated stem cells from discarded adipose tissue after surgeries, the characteristics of which have been reported in our previous study [40].

ADSC-EVs were isolated and purified by ultracentrifugation of cell-free and filtered ADSC cultured media. The purification of primary ADSC-EVs was confirmed by the enrichment of the EV marker CD81, and the absence of the cell markers (GM130, calnexin, and cytochrome-C), and was further confirmed by their round shape in TEM images and their size range by NTA, which matches with other previously reported studies [20,32,40,41].

Many previous studies have reported that stem-cell-derived EVs have angiogenic protein, and some studies showed angiogenic miRNAs [20,32]. The studies showing the presence of angiogenic proteins in EVs have mostly been focused on a solitary factor, despite the fact that EVs may contain numerous other angiogenic proteins. Therefore, in the current study, we investigated 20 angiogenic proteins in ADSC-EVs isolated from two human subjects. Angiogenic protein array revealed the presence of multiple angiogenic proteins in the ADSC-EVs, in agreement with previously reported studies [42,43,44,45]. Our results revealed that IL-8 was present at a greater level compared with the other tested angiogenic proteins, while CCL2 was also present at a relatively high level in ADSC-EVs; both of these have been reported to induce angiogenesis [46,47]. Indeed, a previous study on various cell types demonstrated that CCL2 and IL-8 were associated with the EV membrane more than 50% [43]. Moreover, TIMP-1 and TIMP-2, known to inhibit angiogenesis, were also present in the ADSC-EVs, which is in agreement with previous studies showing that both TIMPs were secreted via EVs (bone marrow stem cells and cancer cells) [45,48,49,50,51,52]. VEGF-D, the strongest inducer of angiogenesis and lymphangiogenesis among VEGFs, was also present in high levels in ADSC-EVs [53,54,55].

EVs are well-known mediators of cell-to-cell communication as a result of their ability to deliver biological materials to recipient cells [25], and it is this property of EVs that has led scientists to exploit EVs as therapeutic agents and drug delivery vehicles [25,56]. Importantly, our results show that ADSC-EVs were internalised into endothelial cells. The migration and proliferation of endothelial cells is crucial for the initiation of angiogenesis from pre-existing vasculature [57]. The internalisation of ADSC-EVs caused endothelial cells to migrate more than untreated cells. Moreover, a previous study reported that IL-8 could induce angiogenesis by directly regulating the survival, proliferation, and migration of endothelial cells [46]. Another study showed that IL-8 promotes the proliferation of human intestinal microvascular endothelial cells and phosphorylation of ERK mediated by CXCR2 [47]. Furthermore, it was reported that CCL2 treatment increases the proliferation and migration of endothelial cells [58,59], and VEGF-D induces the proliferation and migration of endothelial cells, which is vital for angiogenesis [60]. Therefore, the high levels of IL-8, CCL2, and VEGF-D in ADSC-EVs may be responsible for the migration and proliferation of endothelial cells observed in our study.

During angiogenesis, and after the migration and proliferation of endothelial cells, blood vessel formation occurs under suitable conditions and in the presence of certain factors [57]. Therefore, to further investigate the angiogenic potential of ADSC-EVs, we examined the blood-vessel-like formation of endothelial cells in vitro. Our results showed that ADSC-EVs induced a more tube-like formation of endothelial cells compared with the control, which was confirmed by AngioTool analysis. The results showed an increased total vessel length, total number of junctions, and junction density. As mentioned previously, high levels of IL-8, CCL2, and VEGF-D may be responsible for the enhanced tube-like formation of endothelial cells [46,47,53,54,58,59]. Further, we performed the Matrigel plug assay in mice to confirm the in vivo angiogenic effect of ADSC-EVs in a preclinical mouse model. Our results showed that ADSC-EVs increased blood vessel formation in in vivo Matrigel plugs compared with the control material only. Furthermore, these results confirmed that ADSCs in the Matrigel effectively induced angiogenesis, which is in agreement with our previous study [61].

Other proteins, miRNA, or a combination of these may be involved in the induction of angiogenesis in endothelial cells in addition to the proangiogenic proteins reported in the current study. To the best of our knowledge, this study is the first to show the presence of IL-8, CCL2, and VEGF-D in stem-cell-derived EVs, as well as in ADSC-EVs. Although antiangiogenic TIMP-1 and TIMP-2 were present in the ADSC-EVs, they did not affect the promotion of angiogenesis. Although we have shown proangiogenic effects of ADSC-EVs in vitro and in vivo, further preclinical studies in ischaemic models are required to evaluate their clinical potential. Furthermore, the knockdown of proteins will also be important in unravelling the role of particular proteins in ADSC-EVs-mediated angiogenesis.

## 5. Conclusions

The results of the current study reveal the presence of proangiogenic proteins (IL-8, CCL2, and VEGF-D) in human primary ADSC-EVs, and also demonstrate the proangiogenic effect of ADSC-EVs in vitro and in vivo. These findings suggest that ADSC-EVs, which are feasibly isolated from adipose tissue, are a promising therapeutic for ischaemic diseases in the future.

## Data Availability

Data will be made available upon reasonable request.
